# SARS-CoV-2 Sequence Analysis during COVID-19 Case Surge, Liberia, 2021

**DOI:** 10.3201/eid2712.211818

**Published:** 2021-12

**Authors:** Bode Shobayo, Mitali Mishra, Stephen Sameroff, Alexandra Petrosov, James Ng, Alper Gokden, Jane MaCauley, Komal Jain, Courtney Renken, James Tanu Duworko, Moses Badio, Wilhemina Jallah, Lisa Hensley, Thomas Briese, W. Ian Lipkin, Nischay Mishra

**Affiliations:** National Public Health Institute of Liberia, Monrovia, Liberia (B. Shobayo, J. MaCauley);; Partnership for Research on Infectious Diseases in Liberia, Monrovia (B. Shobayo, C. Renken, J.T. Duworko, M. Badio, L. Hensley);; Columbia University, New York, New York, USA (M. Mishra, S. Sameroff, A. Petrosov, J. Ng, A. Gokden, K. Jain, T. Briese, W.I. Lipkin, N. Mishra);; National Institutes of Health, Bethesda, Maryland, USA (C. Renken, L. Hensley);; Ministry of Health, Monrovia (W. Jallah)

**Keywords:** COVID-19, respiratory infections, severe acute respiratory syndrome coronavirus 2, SARS-CoV-2, SARS, coronavirus disease, zoonoses, viruses, coronavirus, variants of concern, Delta variant, virus evolution, Liberia

## Abstract

In June 2021, severe acute respiratory syndrome coronavirus 2 (SARS-CoV-2) cases surged in Liberia. SARS-CoV-2 sequences from patients hospitalized during March–July 2021 revealed the Delta variant was in Liberia in early March and was dominant in June, irrespective of geography. Mutations and deletions suggest multiple SARS-CoV-2 Delta variant introductions.

Before May 2021, Liberia reported <10 coronavirus disease (COVID-19) cases per day among its population of ≈5 million (*1*). Thereafter, case numbers, hospitalizations, and deaths rapidly increased and peaked to >200 cases and 10–15 deaths per day in mid-July 2021 (Appendix Figure 1). To determine whether the rapid case surge was associated with the introduction of severe acute respiratory syndrome coronavirus 2 (SARS-CoV-2) variants of concern or newly emerging variants, we collected nasopharyngeal swab samples from 267 hospitalized patients countrywide during March–July 2021 for high-throughput sequencing.

We collected samples in viral transport media from Bomi, Bong, Grand Cape Mount, Lofa, Margibi, Maryland, Montserrado, and Nimba Counties (Appendix Figure 2). We noted sample collection date and site and sex and median age of patients from whom samples were obtained (Table; Appendix Table). We used Buffer AVL (QIAGEN, https://www.qiagen.com) lysis buffer to extract total nucleic acid and performed PCR by using the Triplex-CII-SARS-Cov-2 rRT PCR assay (*2*). We conducted further high-throughput sequencing on 89/267 (33.3%) samples that had cycle threshold values <33 (Appendix Table). 

**Table:** Characteristics of 77 clinical samples collected before and during COVID-19 case surge that yielded complete SARS-CoV-2 coding genomic sequences, Liberia, 2021

Month collected	Total no. samples	Patient sex, no.	Average age, y (SD)	County	No. samples/ county	SARS-CoV-2 variant, no. of samples/county/mo					
						Delta B.1.617.2	Alpha B.1.1.7	Beta B.1.351	Eta B.1.525	Iota B.1.526	20B other
Mar	4	2M, 2F	39.25 (6.05)	Montserrado	3			1	1	1	
				Bong	1	1					
Apr	11	10M, 1F	42.54 (11.52)	Montserrado	10	4	1	3	2		
				Grand Cape Mount	1			1			
May	18	9M, 9F	40.11 (16.82)	Bong	1	1					
				Margibi	1						
				Montserrado	14	6	3	2	2		1
				Nimba	2						2
Jun	36	13M, 23F	39.22 (18.36)	Lofa	5	5					
				Margibi	1	1					
				Maryland	1	1					
				Montserrado	29	29					
Jul	8	4M, 4F	51.25 ( 9.71)	Margibi	1	1					
				Montserrado	5	5					
				Nimba	2	2					

*Liberia experienced a surge in COVID-19 cases during June 2021. Blank cells indicate no variants detected. COVID-19, coronavirus disease; SARS-CoV-2, severe acute respiratory syndrome coronavirus 2.

To prepare libraries, we used the Kapa Hyperplus Kit (Roche, https://www.roche.com) on first strand cDNA synthesized from 89 RNA samples (*3*), then we enriched for SARS-CoV-2 by using myBaits Custom RNA-Seq Kit (Daicel Arbor Biosciences, https://arborbiosci.com). We sequenced captured libraries on Nextseq 2000 or Nextseq 550 (Illumina, https://www.illumina.com), which yielded 5–8 million 220-bp reads per sample. We mapped reads to a SARS-CoV-2 reference sequence (GenBank accession no. NC_045512) to determine variants (Table; Appendix Table).

Of the 89 RNA samples, 77 (86.5%) yielded complete coding sequences with a minimum depth of ≈15× (GISAID accession nos. EPI_ISL_3547663–705, EPI_ISL_3560291, and EPI_ISL_4232122–52). Using high-throughput sequencing data, we generated consensus fasta sequences of 77 SARS-CoV-2 genomic sequences and further analyzed sequences by using Geneious R10 (https://www.geneious.com), Next-Strain (*4*), and GISAID (*5*).

Among 77 genomes recovered, 4 (5.2%) were Alpha variant (B.1.1.7); 6 (7.8%) were Beta variant (B.1.351); 1 (1.3%) was Iota variant (B.1.526); 6 (7.8%) were Eta variant (B.1.525); and 56 (72.7%) were Delta variant (B.1.617.2) viruses (Table). We identified Delta variant viruses in samples collected in early March and in April and May 2021, from Bong County. Delta variant viruses were co-circulating with Alpha, Beta, Eta, Iota, and other 20B variant viruses in Liberia. All 44 sequences recovered during June–July 2021 were from Delta variant viruses (Table). We used complete polyprotein coding sequences from Liberia, other representative SARS-CoV-2 sequences, and variant reference sequences to create a maximum-likelihood, nucleotide-based phylogenetic tree in MEGA X (*6*) (Figure).

**Figure:**
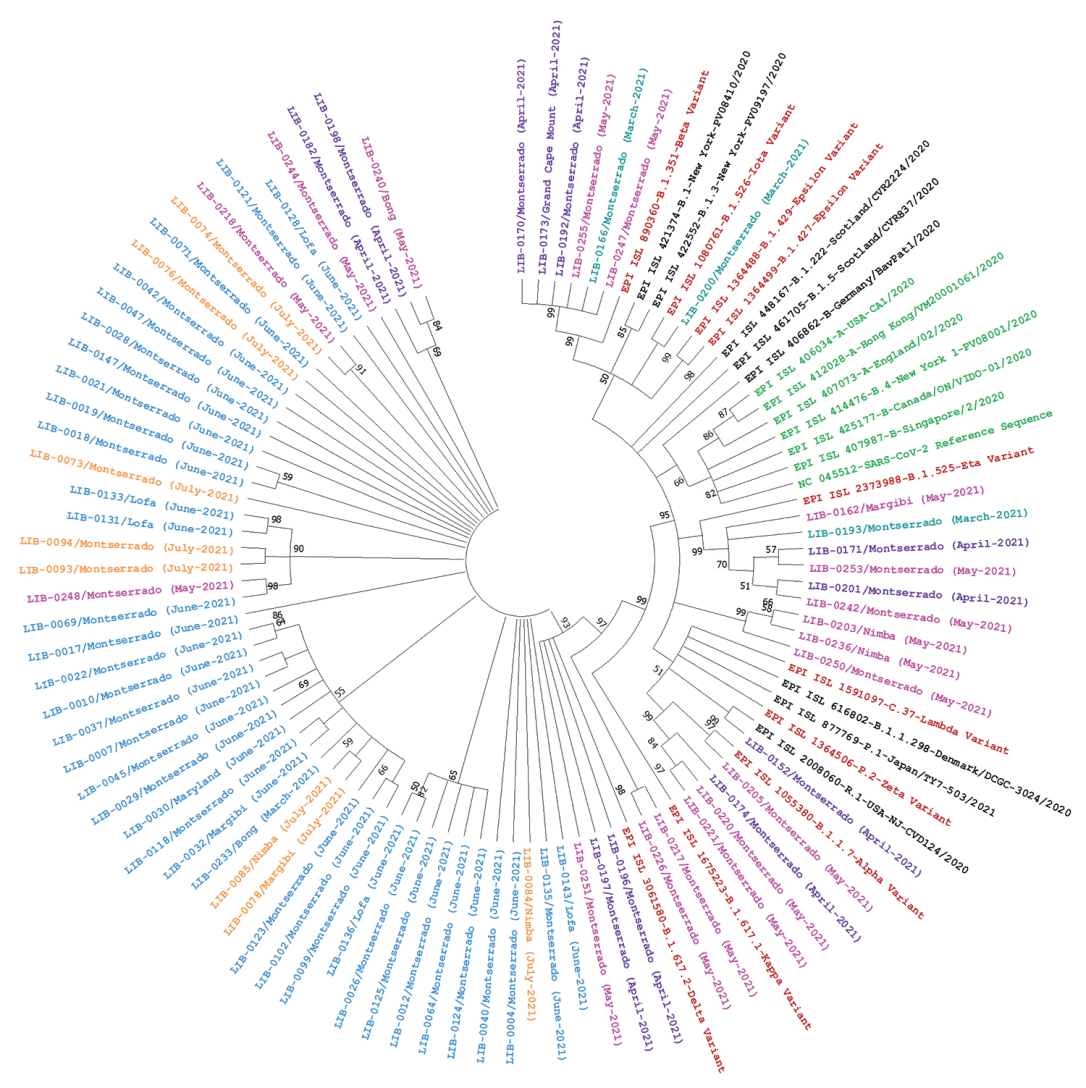
Phylogenetic analysis of 77 nasopharyngeal swab samples collected during coronavirus disease case surge, Libera, March–July 2021, and reference sequences. We created a maximum-likelihood nucleotide phylogenetic tree of the complete polyprotein coding region by using MEGA X (https://www.megasoftware.net), with a bootstrap value of 100 and and used Tamura-Nei 93 (TN93) as a substitution model with a discrete gamma distribution (+G) for evolutionary rate; the rate variation model allowed some sites to be evolutionarily invariable (+I). Numbers along the branches are bootstrap values of 100 bootstrap resamplings. Teal indicates samples collected in March 2021; purple indicates samples collected in April 2021; pink indicates samples collected in May 2021; blue indicates samples collected in June 2021; orange indicates samples collected in July 2021; brown indicates variants of concern or variants of interest; black indicates other circulating variants; green indicates severe acute respiratory syndrome coronavirus 2 reference sequence and other early parental sequences from 2020.

Using reference sequence NC_045512 as a baseline, we found 3 Alpha variant–specific amino acid deletions (H69del, V70del, Y144del) in the surface glycoprotein of all Alpha variant genomes and 3 Beta variant–specific amino acid deletions (L241del, L242del, A243del) in the surface glycoprotein of all Beta variant genomes. All 56 Delta variant genomes had the 2 variant-specific amino acid deletions, F157del and R158del, and 8 of 9 other Delta variant–specific amino acid substitutions in the surface glycoprotein (T19R, G142D, E156G, L452R, T478K, D614G, P681R, and D950N). The A222V surface glycoprotein mutation was absent in only 2/56 Delta variant genomes, LIB-0226 and LIB-0217, collected from Monteserrado County in May 2021 (*4*). We observed another mutation in the surface glycoprotein, V367L, in 14 sequences: 1 from Bong, 2 from Margibi, 1 from Maryland, 9 from Montserrado, and 1 from Nimba. No sequences recovered from Lofa County had the V367L mutation. We noted the R724K mutation in the open reading frame 1a region of 2 sequences from Lofa, LIB-0131 and LIB-0133. LIB-0073 and LIB-0093 sequences collected from Montserrado County had 2 amino acid deletions in the open reading frame 8 region (position 120–121).

Recent surges in COVID-19 in many countries have been associated with the emergence of highly transmissible Delta variant viruses (*7*,*8*). In March 2021, the National Public Health Institute of Liberia sequenced 10 random samples from hospitalized COVID-19 patients in Monteserrado; all sequences were Alpha variant viruses (B. Shobayo, unpub. data). 

A limitation of our study is the small sample sets used for analysis; nonetheless, our findings suggest that Alpha and other circulating variant viruses were replaced by Delta variant viruses countrywide in Liberia in <3 months. Mutation and phylogenetic analyses further indicate that several Delta variant strains were circulating after March 2021 and suggest multiple separate introductions.

Before June 2021, only a small percentage of the population was vaccinated in Liberia. The infections we report occurred in unvaccinated persons. The Ministry of Health, Liberia, initiated a vaccination drive in August 2021. By September, ≈130,000 persons, >2% of the population, had received a single dose of the Johnson & Johnson/Janssen vaccine (https://www.jnj.com). The COVID-19 vaccination campaign is ramping up as <30 cases/day are reported in Liberia, but the currently circulating Delta variants are a concern because they contain mutations and deletions in the surface glycoprotein that might influence vaccine efficacy (*9*). Liberia should continued surveillance for SARS-CoV-2 variants of concern to determine whether additional vaccination or public health measures are needed to curb severe disease and future case surges in the country.

Appendix Additional information on SARS-CoV-2 sequence analysis during COVID-19 case surge, Liberia, 2021.
