# Supplementary material for: SARS-CoV-2 Sequence Analysis during COVID-19 Case Surge, Liberia, 2021
Source: Emerg Infect Dis. 2021 Dec;27(12):3185–8. doi: 10.3201/eid2712.211818 (PMC8632187; doi:10.3201/eid2712.211818)
Supplement: Appendix — Additional information on SARS-CoV-2 sequence analysis during COVID-19 case surge, Liberia, 2021. [file 21-1818-Techapp-s1.pdf]

Article DOI: https://doi.org/10.3201/eid2712.211818

SARS-CoV-2 Sequence Analysis during COVID-19 Case Surge, Liberia, 2021

Appendix

Appendix Table. Dates, collection sites, sex, age, quantitative PCR cycle threshold values, and sequence coverage for 89 samples from COVID-19 patients, Liberia, 2021

Sample ID	Collection date	County	Age, y/sex	C_t value	% Genome recovered	Avg. depth/nucleotide	Nextclade	Variant†	GISAID clade
LIB-0233	9 Mar	Bong	47/M	32.39	99.82	53.35	21A	VOC Delta B.1.617.2	G
LIB-0193	10 Mar	Montserrado	30/F	32.70	99.73	29.48	21D	VOI Eta B.1.525	G
LIB-0166	31 Mar	Montserrado	40/F	26.94	99.94	1534.63	20H	VOC Beta B.1.351	GH
LIB-0200	31 Mar	Montserrado	40/M	23.43	99.94	14633.94	21F	VOI Iota B.1.526	GH
LIB-0170	2 Apr	Montserrado	58/M	24.77	99.97	24611.29	20H	VOC Beta B.1.351	GH
LIB-0171	4 Apr	Montserrado	64/F	25.04	99.86	185.61	21D	VOI Eta B.1.525	G
LIB-0174	4 Apr	Montserrado	35/M	24.63	99.98	27842.07	20I	VOC Alpha B.1.1.7	GR
LIB-0192	4 Apr	Montserrado	30/M	22.07	100.00	69811.5	20H	VOC Beta B.1.351	GH
LIB-0197	6 Apr	Montserrado	54/M	23.87	99.96	16087.41	21A	VOC Delta B.1.617.2	G
LIB-0152	8 Apr	Montserrado	41/M	24.83	99.91	681.27	20B	Other	GR
LIB-0173	8 Apr	Grand Cape Mount	41/M	27.23	99.94	2708	20H	VOC Beta B.1.351	GH
LIB-0196	10 Apr	Montserrado	30/M	19.29	100.00	93864.59	21A	VOC Delta B.1.617.2	G
LIB-0198	10 Apr	Montserrado	27/M	24.05	99.98	11960.47	21A	VOC Delta B.1.617.2	G
LIB-0201	13 Apr	Montserrado	46/M	26.42	99.94	2851.87	21D	VOI Eta B.1.525	G
LIB-0182	17 Apr	Montserrado	42/M	23.72	99.99	22901.55	21A	VOC Delta B.1.617.2	G
LIB-0204	3 May	Montserrado	20/M	32.16	43.18	1.62	NA	NA	NA
LIB-0220	3 May	Montserrado	34/M	29.37	99.92	215.57	20I	VOC Alpha B.1.1.7	GR
LIB-0248	4 May	Montserrado	30/M	31.31	99.85	100.86	21A	VOC Delta B.1.617.2	G
LIB-0206	15 May	Montserrado	46/M	28.51	57.74	1.99	NA	NA	NA
LIB-0162	17 May	Margibi	77/M	32.11	99.85	434.73	21D	VOI Eta B.1.525	G
LIB-0236	17 May	Nimba	62/F	23.46	99.94	4894.08	20B	Other	GR
LIB-0203	20 May	Nimba	34/M	32.15	99.71	39.59	20B	Other	GR
LIB-0205	20 May	Montserrado	64/F	31.14	99.88	64.74	20I	VOC Alpha B.1.1.7	GR
LIB-0217	20 May	Montserrado	37/M	30.01	99.89	175.39	21A	VOC Delta B.1.617.2	G
LIB-0218	20 May	Montserrado	24/F	32.81	99.81	18.65	21A	VOC Delta B.1.617.2	G
LIB-0221	20 May	Montserrado	10/F	31.91	99.71	14.87	20I	VOC Alpha B.1.1.7	GR
LIB-0225	20 May	Montserrado	68/M	32.97	85.36	2.78	NA	NA	NA
LIB-0226	20 May	Montserrado	63/M	32.64	99.80	34.1	21A	VOC Delta B.1.617.2	G

Sample ID	Collection date	County	Age, y/sex	C_t value	% Genome recovered	Avg. depth/nucleotide	Nextclade	Variant†	GISAID clade
LIB-0247	20 May	Montserrado	43/F	18.86	100.00	303638.25	20H	VOC Beta B.1.351	GH
LIB-0250	20 May	Montserrado	38/F	22.72	99.98	14212.06	20B	Other	GR
LIB-0251	20 May	Montserrado	33/F	26.09	100.00	7626.71	21A	VOC Delta B.1.617.2	G
LIB-0255	20 May	Montserrado	31/M	17.25	100.00	504226.47	20H	VOC Beta B.1.351	GH
LIB-0253	27 May	Montserrado	27/F	18.58	100.00	223902.08	21D	VOI Eta B.1.525	G
LIB-0240	29 May	Bong	54/M	16.01	100.00	193424.09	21A	VOC Delta B.1.617.2	G
LIB-0242	29 May	Montserrado	23/F	22.93	99.99	18571.69	21D	VOI Eta B.1.525	G
LIB-0244	29 May	Montserrado	38/M	19.90	100.00	15944.35	21A	VOC Delta B.1.617.2	G
LIB-0004	5 Jun	Montserrado	57/M	21	100.00	132105.37	21A	VOC Delta B.1.617.2	G
LIB-0007	5 Jun	Montserrado	67/F	21	100.00	24117.24	21A	VOC Delta B.1.617.2	G
LIB-0071	5 Jun	Montserrado	28/M	28	99.98	748.95	21A	VOC Delta B.1.617.2	G
LIB-0017	6 Jun	Montserrado	62/F	28	99.94	302.55	21A	VOC Delta B.1.617.2	G
LIB-0018	6 Jun	Montserrado	41/M	24	99.30	106.19	21A	VOC Delta B.1.617.2	G
LIB-0019	6 Jun	Montserrado	34/M	17	99.90	359124.54	21A	VOC Delta B.1.617.2	G
LIB-0021	6 Jun	Montserrado	56/F	22	100.00	24466.47	21A	VOC Delta B.1.617.2	G
LIB-0022	6 Jun	Montserrado	37/F	25	100.00	1307.05	21A	VOC Delta B.1.617.2	G
LIB-0026	6 Jun	Montserrado	38/M	19	99.90	366820.93	21A	VOC Delta B.1.617.2	G
LIB-0028	6 Jun	Montserrado	27/F	27	99.94	797.2	21A	VOC Delta B.1.617.2	G
LIB-0032	6 Jun	Margibi	46/F	23	100.00	30961.5	21A	VOC Delta B.1.617.2	G
LIB-0064	8 Jun	Montserrado	40/F	28	99.95	399.9	21A	VOC Delta B.1.617.2	G
LIB-0012	12 Jun	Montserrado	45/F	19	100.00	12751.38	21A	VOC Delta B.1.617.2	G
LIB-0029	12 Jun	Montserrado	30/M	23	100.00	6319.4	21A	VOC Delta B.1.617.2	G
LIB-0030	12 Jun	Maryland	32/F	25	100.00	7395.49	21A	VOC Delta B.1.617.2	G
LIB-0037	12 Jun	Montserrado	35/M	20	100.00	2987.68	21A	VOC Delta B.1.617.2	G
LIB-0040	12 Jun	Montserrado	38/M	22	100.00	40117.32	21A	VOC Delta B.1.617.2	G
LIB-0042	12 Jun	Montserrado	29/M	22	100.00	23365.37	21A	VOC Delta B.1.617.2	G
LIB-0045	12 Jun	Montserrado	32/F	20	100.00	10116.23	21A	VOC Delta B.1.617.2	G
LIB-0047	12 Jun	Montserrado	32/F	22	100.00	46601.79	21A	VOC Delta B.1.617.2	G
LIB-0128	15 Jun	Lofa	1/M	21	100.00	29163.85	21A	VOC Delta B.1.617.2	G
LIB-0010	16 Jun	Montserrado	26/F	24	100.00	9787.07	21A	VOC Delta B.1.617.2	G
LIB-0131	16 Jun	Lofa	56/F	25	100.00	1864.44	21A	VOC Delta B.1.617.2	G
LIB-0133	16 Jun	Lofa	47/F	26	99.99	65698.71	21A	VOC Delta B.1.617.2	G
LIB-0136	16 Jun	Lofa	35/M	22	99.98	1442.69	21A	VOC Delta B.1.617.2	G
LIB-0137	16 Jun	Bomi	40/F	25	0	NA	NA	NA	NA
LIB-0143	16 Jun	Lofa	30/F	23	99.95	5513	21A	VOC Delta B.1.617.2	G

Sample ID	Collection date	County	Age, y/sex	C_t value	% Genome recovered	Avg. depth/nucleotide	Nextclade	Variant†	GISAID clade
LIB-0118	17 Jun	Montserrado	41/M	19	100.00	74762.42	21A	VOC Delta B.1.617.2	G
LIB-0121	17 Jun	Montserrado	82/F	30	99.66	40	21A	VOC Delta B.1.617.2	G
LIB-0123	17 Jun	Montserrado	25/F	21	100.00	10448.21	21A	VOC Delta B.1.617.2	G
LIB-0135	17 Jun	Montserrado	55/F	23	99.97	598.15	21A	VOC Delta B.1.617.2	G
LIB-0099	18 Jun	Montserrado	2/F	21	100.00	12031.71	21A	VOC Delta B.1.617.2	G
LIB-0124	18 Jun	Montserrado	25/F	29	99.88	117.63	21A	VOC Delta B.1.617.2	G
LIB-0125	18 Jun	Montserrado	89/F	22	100.00	13008.5	21A	VOC Delta B.1.617.2	G
LIB-0102	19 Jun	Montserrado	8/F	20	100.00	10170.05	21A	VOC Delta B.1.617.2	G
LIB-0130	24 Jun	Montserrado	37/M	30	71.32	NA	NA	NA	NA
LIB-0134	24 Jun	Montserrado	62/F	28	12.74	NA	NA	NA	NA
LIB-0142	26 Jun	Montserrado	42/F	27	5.53	NA	NA	NA	NA
LIB-0147	26 Jun	Montserrado	36/M	30	99.97	1970.45	21A	VOC Delta B.1.617.2	G
LIB-0069	27 Jun	Montserrado	48/F	19	100.00	86159.99	21A	VOC Delta B.1.617.2	G
LIB-0111	7 Jul	Montserrado	11/F	29	0.65	0.9	NA	NA	NA
LIB-0113	7 Jul	Montserrado	48/M	26	1.65	0.86	NA	NA	NA
LIB-0073	8 Jul	Montserrado	31/F	29	99.47	117.79	21A	VOC Delta B.1.617.2	G
LIB-0074	8 Jul	Montserrado	47/M	24	100.00	4168.52	21A	VOC Delta B.1.617.2	G
LIB-0076	8 Jul	Montserrado	51/F	22	100.00	14232.36	21A	VOC Delta B.1.617.2	G
LIB-0078	8 Jul	Margibi	54/F	23	100.00	12901.34	21A	VOC Delta B.1.617.2	G
LIB-0093	8 Jul	Montserrado	48/M	27	99.31	84.44	21A	VOC Delta B.1.617.2	G
LIB-0103	8 Jul	Montserrado	41/F	29	0	NA	NA	NA	NA
LIB-0112	8 Jul	Montserrado	32/F	29	0.67	1.67	NA	NA	NA
LIB-0084	9 Jul	Nimba	67/M	27	99.91	1228.28	21A	VOC Delta B.1.617.2	G
LIB-0085	9 Jul	Nimba	59/M	22	100.00	63134.43	21A	VOC Delta B.1.617.2	G
LIB-0094	10 Jul	Montserrado	52/F	32	99.93	1979.92	21A	VOC Delta B.1.617.2	G
LIB-0101	20 Jul	Montserrado	64/M	31	26.44	NA	NA	NA	NA

*Twelve samples had insufficient genome coverage of average 15× depth/nucleotide.C_t, cycle threshold; NA, not applicable; VOC, variant of concern; VOI, variant of interest. Sequencing data can be accesed using GISAID (https://www.gisaid.org) accession numbers EPI_ISL_3547663–705, EPI_ISL_3560291, and EPI_ISL_4232122–52).

†According to Phylogenetic Assignment of Named Global Outbreak Lineages (https://cov-lineages.org) software tool.

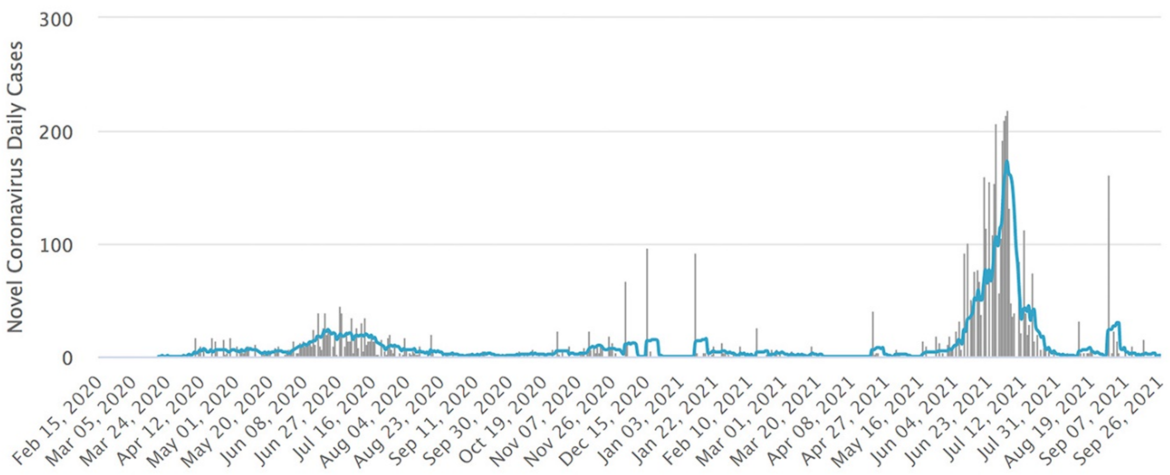

Appendix Figure 1. Daily coronavirus disease cases, Liberia, February 2020–September 2021. Blue lines indicate 7 day moving average of daily new cases. Data were derived from Worldometer (https://www.worldometers.info/coronavirus/country/liberia).

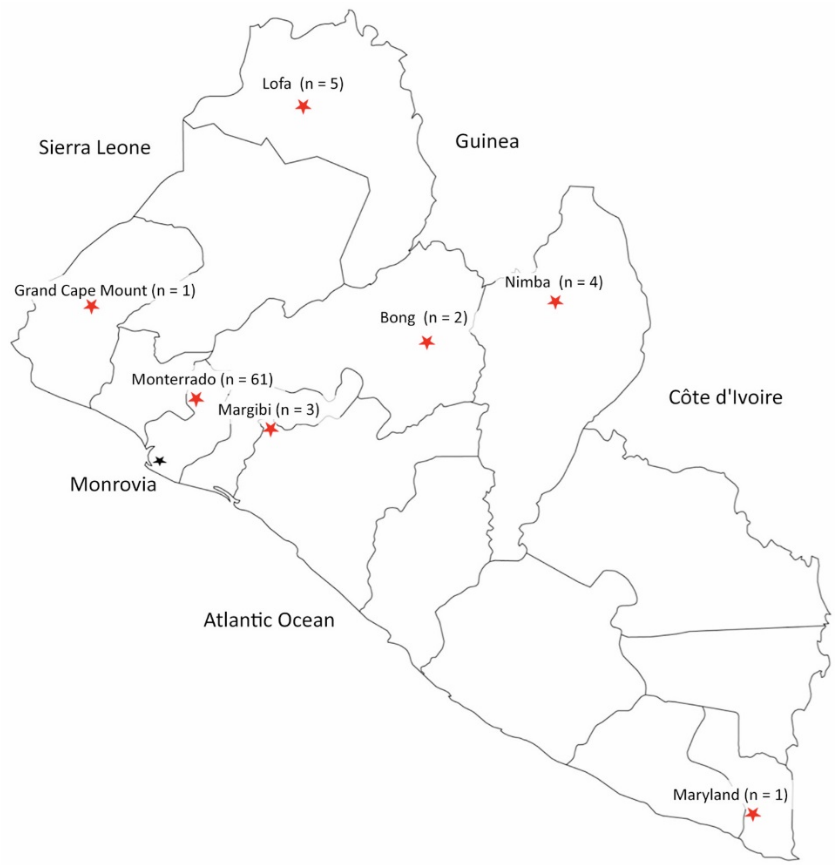

Appendix Figure 2. Sites from which nasopharyngeal swab samples were collected during a coronavirus disease case surge, Liberia, 2021. Red stars indicate locations inside counties; black star indicates the capital, Monrovia. Numbers of cases per county are indicated in parentheses.
